# Impact of immediate-release versus extended-release metformin on lipid profile and body measurements in Saudi patients with type 2 diabetes: a prospective cohort study

**DOI:** 10.11604/pamj.2025.52.176.49165

**Published:** 2025-12-19

**Authors:** Hisham Alshadfan, Hyder Mirghani, Tariq Alrasheed, Mansuor Alanazi, Muhammad Nazrul Hakim Abdullah

**Affiliations:** 1 Department of Biomedical Sciences, Faculty of Medicine and Health Sciences, University Putra Malaysia, Serdang, Selangor, Malaysia,; 2 Department of Clinical Biochemistry, Faculty of Medicine, University of Tabuk, Tabuk, Saudi Arabia,; 3 Department of Internal Medicine, Faculty of Medicine, University of Tabuk, Tabuk, Saudi Arabia,; 4 Department of Family and Community Medicine, Faculty of Medicine, University of Tabuk, Tabuk, Saudi Arabia,; 5 Halal Product Institute, Universiti Putra Malaysia, Serdang, Selangor, Malaysia

**Keywords:** Type 2 diabetes, metformin immediate-release, metformin extended-release, lipid profile, body measurements

## Abstract

**Introduction:**

dyslipidemia and obesity frequently accompany type 2 diabetes, contributing to cardiovascular risk. Metformin, beyond its effects on glycemic control, can also influence lipid metabolism and weight. Immediate-release (IR) and extended-release (XR) metformin may have differential effects on lipid profile and anthropometric measures. The study aimed to compare the effects of metformin IR versus XR on lipid parameters and body measurements in patients with type 2 diabetes in Saudi Arabia.

**Methods:**

a prospective 6-month cohort study included 119 newly diagnosed type 2 diabetes patients (62 on metformin IR and 57 on metformin XR) at a Saudi hospital. Fasting lipid profile (T-Chol, TGs, HDL-C, and LDL-C) and body weight/BMI were measured at baseline and after 6 months of metformin treatment. Within-group changes and between-group differences were analysed.

**Results:**

both groups showed modest improvements in their lipid profile. Metformin XR use was associated with a greater reduction in total cholesterol (-7.8% vs. -5.8% with IR) and LDL-C (-8.7% vs. -5.7% with IR). After 6 months, mean LDL-C was significantly lower in the XR group (102.3 ± 37.5 mg/dL) compared to the IR group (116.9 ± 36.0 mg/dL, p=0.032). Triglyceride and HDL-C levels changed minimally and were similar between groups. Neither IR nor XR had a significant impact on body weight or BMI: both groups remained essentially weight-neutral (average weight change <1 kg over 6 months). Final mean BMI was 30.4 ± 5.9 kg/m^2^ in IR vs. 29.8 ± 4.7 kg/m^2^ in XR (no significant difference).

**Conclusion:**

metformin XR was associated with short-term benefits in reducing total and LDL cholesterol levels compared to IR, likely attributable to more stable plasma drug concentrations that enhance lipid metabolism. Both formulations exhibited similar effects on triglycerides, HDL cholesterol, and body weight, reaffirming metformin's overall neutral impact on weight. These findings suggest that metformin XR may offer a marginal reduction in cardiovascular risk through superior LDL cholesterol lowering, while preserving the weight-neutral profile characteristic of metformin therapy.

## Introduction

Type 2 diabetes is commonly accompanied by a cluster of metabolic abnormalities, including dyslipidemia and excess weight. Diabetic dyslipidemia typically features elevated triglycerides and low HDL cholesterol, along with qualitatively altered LDL particles, all of which heighten cardiovascular disease risk [1,2]. Weight gain or difficulty losing weight also complicates diabetes management and risk stratification. Therefore, an ideal antidiabetic therapy would improve glycemic control and favorably influence lipid profile and weight, or at least be weight-neutral.

Metformin, a first-line oral agent for type 2 diabetes, is generally considered weight-neutral or associated with modest weight loss, in contrast to insulin or sulfonylureas. It has also been documented to have beneficial effects on serum lipids, although these effects are moderate [3,4]. Mechanistically, metformin's activation of AMP-activated protein kinase (AMPK) in the liver leads not only to reduced gluconeogenesis but also to decreased lipogenesis and enhanced lipid oxidation. These actions can result in lower circulating cholesterol and triglyceride levels [5,6]. Some studies have indeed observed reductions in total and LDL cholesterol with metformin therapy. For example, Wulffelé *et al*. reported that metformin improved the lipid profile by lowering LDL-C and triglycerides while raising HDL-C in diabetic patients [7]. However, not all studies consistently show lipid changes, and the magnitude is usually modest (e.g., 5-10% reductions) compared to potent lipid-specific drugs. Metformin is available in immediate-release (IR) and extended-release (XR) formulations. The XR formulation provides slower, more gradual absorption, which might confer advantages in metabolic effects beyond glucose. One question is whether XR metformin has different or stronger effects on lipids or weight than IR metformin. The rationale is that steadier metformin levels (with XR) might sustain AMPK activation and lipid-lowering effects over 24 hours. In contrast, IR's peaks and troughs could be less effective in that regard [5,8]. Some evidence suggests differences: a 2021 systematic review found that patients on metformin XR had significantly lower LDL cholesterol levels (~5.7 mg/dL lower) compared to those on IR across trials [9]. This suggests XR could have a slight edge in improving LDL levels. On the other hand, many clinical trials, such as Aggarwal *et al*. and others, reported no significant differences in lipid outcomes between XR and IR over 4-6 months [10]. For body weight, meta-analyses have consistently shown that metformin does not cause weight gain; immediate versus extended formulations appear to have a similar impact on weight [11,12]. Abrilla *et al*. noted no significant difference in weight change between IR and XR in their meta-analysis [13]. Both formulations generally produce a slight weight reduction or stabilisation in patients, likely due to metformin's effect of reducing hepatic fat and possibly suppressing appetite.

Despite these insights, data specifically focusing on lipid and anthropometric outcomes in Middle Eastern populations are scarce. Saudi patients with T2DM often have a high prevalence of obesity and dyslipidemia. Understanding how metformin formulations affect these parameters could inform therapy choices that address cardiovascular risk. Our study aims to directly compare the effects of metformin IR versus XR on the lipid profile (total cholesterol, LDL-C, HDL-C, and triglycerides) and body measurements (weight and BMI) over six months in a Saudi cohort. We hypothesise that metformin XR may lead to greater improvements in atherogenic lipid parameters due to more continuous drug exposure, while both formulations will be similarly weight-neutral. Confirming even minor differences could have implications for managing dyslipidemia in patients with diabetes.

## Methods

**Study design, setting, and participants:** this was a 6-month prospective cohort study conducted at the diabetes centre of King Fahad Specialist Hospital, Tabuk, Saudi Arabia. The design involved two parallel patient groups: one receiving immediate-release (IR) metformin therapy and one receiving extended-release (XR) metformin, both observed under real-world conditions. A total of 119 newly diagnosed type 2 diabetes patients were followed (62 in the IR group, 57 in the XR group). Patients were allocated to IR or XR by their treating physicians at the time of diabetes diagnosis (not randomised by the investigators).

**Study duration:** patient recruitment and baseline data collection took place from March 2023 to June 2024, with follow-up evaluations completed by December 2024. Each patient was observed for around 6 months, starting from enrollment (when metformin therapy began) until the final follow-up. The entire study lasted approximately 21 months, allowing for staggered enrollment and ensuring that all patients completed their 6-month follow-up by the end of 2024.

**Study population and sampling:** the target group consisted of newly diagnosed adult patients with type 2 diabetes in Tabuk, Saudi Arabia. “Newly diagnosed” refers to patients diagnosed within the last 3 months who had not yet started any antidiabetic medication except metformin. All participants were Saudi nationals (to ensure a uniform ethnic sample), aged between 18 and 75. Both men and women were eligible. We specifically included drug-naïve individuals to accurately assess the effects of metformin IR versus XR without interference from other glucose-lowering medications.

Systematic random sampling was used to select eligible patients from new referrals at the diabetes centre. The clinic records indicated that about 4-8 new patients with diabetes were seen weekly. With an estimated total of around 480 new cases during the recruitment period and a target sample size of approximately 142, a sampling interval (k) of 3 was determined (selecting roughly every 3^rd^ patient). A random start between 1 and 3 was chosen, and we selected the second patient (using a random digit table). This meant enrolling the second eligible patient, then every third patient thereafter, as patients were sequentially assigned to IR or XR by their physician. If a patient declined or was ineligible, the next eligible patient was enrolled to maintain the sequence. This method helped ensure an unbiased sample of the clinic population starting metformin treatment and balanced the groups. Ultimately, the group assignment was based on the prescribed formulation, but our systematic approach minimised selection bias and captured typical patients on each regimen. Some patients were prescribed the immediate-release (IR) formulation, while others received the extended-release (XR) formulation, based on the physician's judgment. These existing prescribing practices informed the formation of our two study groups: the Metformin IR group, which includes patients provided immediate-release metformin at diagnosis with a target dose around 1500 mg/day divided into three doses, and the Metformin XR group, which includes patients provided extended-release metformin at diagnosis with a target dose roughly 1500 mg/day, often taken once daily or divided into two doses.

### Inclusion and exclusion criteria

**Inclusion criteria:** eligible participants were Saudi adults aged 18 to 75 who had been newly diagnosed with type 2 diabetes, as defined by ADA criteria, within the preceding three months. They needed to provide informed consent and have an initial HbA1c of 6.5% to 8.5%, indicating mild to moderate hyperglycemia suitable for metformin monotherapy. Only those starting metformin, either immediate- or extended-release, as their initial treatment without prior exposure to other glucose-lowering drugs, were included. Participants also had to agree to complete the six-month follow-up visits and required evaluations.

**Exclusion criteria:** participants were excluded if they had conditions likely to affect study outcomes or prevent the safe use of metformin. This included having type 1 diabetes or needing insulin at diagnosis, significant diabetes-related complications such as advanced kidney disease or proliferative retinopathy, or other serious uncontrolled chronic illnesses. Individuals with a prior history of lactic acidosis or contraindications to metformin, such as severe renal or liver impairment or alcohol misuse, were excluded, as were women who were pregnant, breastfeeding, or planning pregnancy, to avoid metabolic variability and drug exposure risks. Those with severe gastrointestinal disorders or previous intolerance to metformin, as well as individuals with cognitive or psychiatric conditions that could hinder consent or adherence, were not eligible. Only Saudi nationals were included to minimise genetic and lifestyle variation, and anyone meeting the criteria but declining participation was not enrolled.

**Figure 1:**
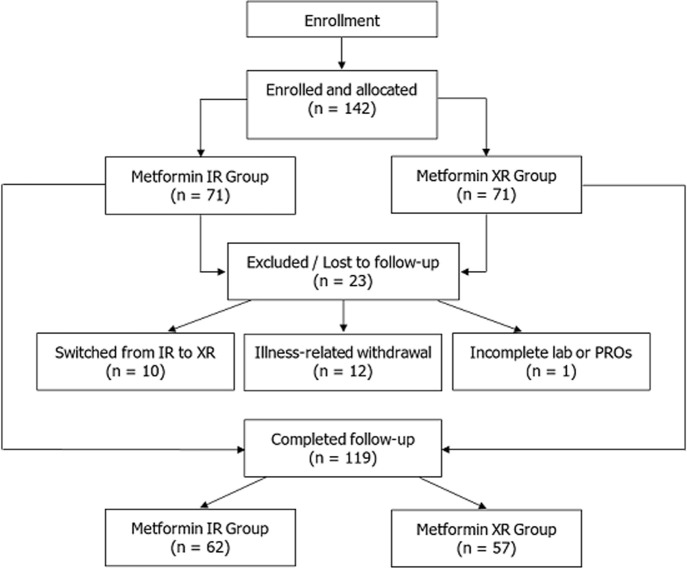
participant flow diagram showing enrollment, group allocation, follow-up, and reasons for exclusion

**Sample size:** the sample size was determined to detect a clinically relevant difference in HbA1c reduction between treatment arms. Based on a two-tailed comparison of means, 80% power, and a 0.05 significance level, approximately 59 participants per group were required to detect a 0.5% HbA1c difference, assuming a standard deviation of 1.3% from prior studies. Allowing for as much as 20% attrition, the recruitment goal was set at 71 individuals per group (142 total). Ultimately, 119 participants completed the trial (62 on IR and 57 on XR), yielding an 83.8% response rate, which reflects a combination of biological, social, and cultural factors that contributed to strong participant engagement (Figure 1).

Although the original sample size was based on HbA1c differences, a post-hoc power analysis using a two-sample t-test showed that the final sample (n=119) provided approximately 80% power to detect the observed between-group difference in LDL-C (15 mg/dL, pooled SD~36.8 mg/dL, α=0.05), supporting the validity of the lipid outcome analysis.

**Data collection and measurements:** at baseline and 6-month follow-up, data were collected through patient interviews, clinical measurements, and lab tests. Specifically, for this analysis: lipid profile: fasting blood samples (after a 10-12 hour fast) were collected to measure total cholesterol (T-Chol), triglycerides (TG), high-density lipoprotein cholesterol (HDL-C), and low-density lipoprotein cholesterol (LDL-C). Standard enzymatic assays were performed on an automated analyser. LDL-C was calculated by the Friedewald formula for TG <400 mg/dL or measured directly if higher. Body measurements: body weight (kg) was measured using a calibrated digital scale, with participants in light clothing and no shoes. Height (m) was recorded with a stadiometer. Body mass index (BMI) was calculated as weight (kg) / height (m^2^). We also noted waist circumference in the full data, but here we focus on weight and BMI as primary anthropometric outcomes. Other data: baseline demographics, glycemic indices (reported in companion paper), and any medication adjustments were recorded. Importantly, patients were asked about any diet or lifestyle changes that could affect weight or lipids, to be considered in context. All participants received similar standard-of-care advice on diet (a balanced diabetic diet) and exercise, regardless of the metformin formulation.

The same report titled “Socio-demographic, Body Measurements, Laboratory Parameters, and GI Adverse Events Report (SBLG-R)” and the methodology described earlier were used to ensure consistency in data gathering. By design, none of the patients were on lipid-lowering medications (statins) at baseline, as they were newly diagnosed (if any indicated statin therapy, it was noted if started during follow-up). In practice, a few patients with a baseline LDL level > 130 mg/dL were advised to undergo lifestyle modification first; none started statins during the short follow-up period unless their LDL levels were extremely high (we excluded any patients who initiated a statin, as this would confound the results).

**Study endpoints:** changes in lipid profile (total cholesterol, triglycerides, HDL-C, and LDL-C) were defined as the absolute difference in concentration (mg/dL) between baseline and 6-month follow-up values. Furthermore, changes in body measurements were similarly defined as the absolute difference in weight (kg) and body mass index (kg/m^2^) between baseline and follow-up. These absolute differences were used in all between-group and within-group analyses.

**Statistical analysis:** normality was assessed using multiple criteria: visual inspection of histograms and Q-Q plots, skewness and kurtosis z-scores (±2.58 threshold), and the Shapiro-Wilk test (p>0.05 indicating normality). A variable was considered normally distributed only if at least three of the four criteria were met at both baseline and 6-month follow-up. This guided the selection of parametric or non-parametric tests.

To test our hypothesis that metformin XR would lead to greater improvements in atherogenic lipid parameters while both formulations would have a similar (neutral) effect on body weight and BMI, we conducted between-group comparisons of change scores over the 6 months. To compare the lipid profile (T-Chol, TG, HDL-C, and LDL-C) and body measurements (body weight and BMI) between the metformin IR and XR groups, we used independent sample t-tests for normally distributed data and Mann-Whitney U tests for non-normally distributed data. Within each group (baseline and 6-month), values were compared using paired-sample t-tests or Wilcoxon signed-rank tests. (based on normality tests performed). Normally distributed data were analysed using parametric tests and presented as mean ± standard deviation. Non-normally distributed data were analysed using non-parametric tests and summarised with medians (interquartile ranges). Additionally, categorical outcomes were compared with chi-square tests. A p<0.05 was considered significant. All analyses were done with SPSS (version 29.0). Graphical representations (bar charts) were created for key outcomes. No imputation was performed for missing data; only patients with both baseline and 6-month values were included (which was > 95% of participants for lipid and weight measures).

**Ethical clearance:** the study protocol was reviewed and approved by the relevant ethical committees the Local Research Ethics Committee of the University of Tabuk, Saudi Arabia (Approval No. UT-190-46-2022; 16^th^ March 2022), Tabuk Institutional Review Board of the Ministry of Health, Tabuk, Saudi Arabia (Protocol No. TU-077/022/137; 31^st^ May 2022), and the Ethics Committee for Research Involving Human Subjects of *Universiti Putra Malaysia* (JKEUPM) (Reference No. JKEUPM-2022-860; 7^th^ March 2023). All participants provided informed consent, and their confidentiality was protected. The study posed minimal risk; permission included analysing laboratory parameters, such as lipid profile, and conducting body measurements as part of routine care. Ethical oversight ensured that any abnormal lipid results were communicated to patients and managed according to clinical guidelines (e.g., dietary counselling, consideration of therapy outside the study).

## Results

**General characteristics of participants:** a total of 119 patients were included in the study (average age approximately 50.3 ± 8.7 years), with 62 on metformin IR and 57 on metformin XR. The group comprised 47% males and 53% females, representing the typical patient population.

**Baseline characteristics of IR and XR groups:** there were no significant statistical differences between metformin IR and metformin XR groups regarding gender: 50% versus 43.9% were males, and 50% versus 56.1% were females, with no statistically significant differences (X2=0.45, p=0.503). The mean age of the IR group was 49.9 ± 8.5 years, compared to 50.6 ± 9.0 years for the XR group, with no statistically significant difference between the groups (MD=-0.73, -3.90 - 2.45 95%CI, t=-0.45, p=0.65). The median duration of diabetes was 3.0 (1.0) months, with no statistically significant difference between the two groups (Z=-0.51, p=0.612) (Table 1).

**Table 1:** baseline characteristics of metformin IR and XR groups

Variable	Metformin IR n=62	Metformin XR n=57	MD	95% CI	X2/t/Z	P-value
Gender						
**Male**	31 (50.0)	25 (43.9)			0.45**	**0.503**
**Female**	31 (50.0)	32 (56.1)				
Age (years)	49.9 ± 8.5	50.6 ± 9.0	-0.73	-3.90 2.45	-0.45	**0.650**
Duration of diabetes (months)	3.0 (1.0)	3.0 (1.0)			-0.51*	**0.612**
Lipid profile						
**T-Chol (mg/dL)**	202.4 ± 54.2	187.7 ± 43.6	14.77	-3.18 32.72	1.63	**0.106**
**TGs (mg/dL)**	160.0 (84.8)	121.0 (110.0)			-0.50*	**0.617**
**HDL-C (mg/dL)**	41.0 (8.5)	43.0 (8.0)			-0.41*	**0.685**
**LDL-C (mg/dL)**	124.0 ± 47.0	112.1 ± 43.2	11.83	-4.54 28.21	1.43	**0.156**
Body measurements						
**Body weight (kg)**	82.9 ± 17.74	84.6 ± 12.8	-1.71	-7.36 3.94	-0.60	**0.550**
**BMI (kg/m^2^)**	**30.9 ± 5.74**	**30.0 ± 4.6**	**0.86**	**-1.04 2.77**	**0.90**	**0.370**

Significant level at P<0.05; an independent sample t-test was adopted in this statistical analysis for parametric data with mean ± SD; * the Mann-Whitney U test was adopted in this statistical analysis for non-parametric data with median (IQR); ** Pearson’s chi-square test was adopted in this statistical analysis for categorical data with N (%)

**Baseline lipid and weight profiles:** the IR and XR groups had comparable lipid profiles at baseline. Mean total cholesterol was 202.4 ± 54.2 mg/dL in the IR group and 187.7 ± 43.6 mg/dL in the XR group (MD=14.77, -3.18 - 32.72 95%CI, t=1.63, p=0.106). The median triglycerides were slightly elevated but not extreme (IR: 160.0 (84.8) mg/dL, XR: 121.0 (110.0) mg/dL, Z=-0.5, p=0.617). The median HDL-C levels were low in both groups (IR: 41.0 (8.5) mg/dL, XR: 43.0 (8.0) mg/dL, p=0.685). LDL-C averages were similarly high-normal: IR 124.0 ± 47.0 mg/dL vs. XR 112.1 ± 43.2 mg/dL (Z=-0.41, p=0.156) (Table 1). Thus, both cohorts showed the typical dyslipidemic pattern of T2DM with no significant differences between them initially.

In terms of anthropometrics, both groups were overweight/obese on average. Baseline mean weight was 82.9 ± 17.7 kg (IR) vs. 84.6 ± 12.8 kg (XR) (MD=-1.71, -7.36 - 3.94 95%CI, t=-0.6, p=0.55), and mean BMI was 30.9 ± 5.7 kg/m^2^ vs. 30.0 ± 4.6 kg/m^2^, respectively (MD=0.86, -1.04 - 2.77 95%CI, t=0.9, p=0.37) (Table 1). The distribution of BMI categories was similar: approximately 53% of participants in each group were obese (BMI ≥30), around 33% were overweight (BMI 25-29.9), and only about 14% had a normal BMI. No significant differences in baseline weight or BMI were observed, aligning with the findings that our systematic sampling was associated with comparable groups.

**Changes in lipid profile after 6 months:** metformin therapy was associated with a trend toward an improved lipid profile in both groups; however, changes were more pronounced in the XR group for certain parameters, particularly total and LDL cholesterol. i) Total cholesterol: in the IR group, the mean total cholesterol showed a slight decrease, from 202.4 ± 54.2 to 190.7 ± 41.8 mg/dL over 6 months (mean change, -11.7 mg/dL or -5.8%, -7.36 - 3.94 95%CI, t=-0.6, p=0.014) (Table 2). The XR group experienced a greater decline, from 187.7 ± 43.6 to 173.0 ± 40.0 mg/dL (mean change, -14.7 mg/dL or -7.8%, 7.45 - 21.92 95%CI, t=4.07, p<0.001) (Table 3). After 6 months, the average T-Chol in XR patients was ~18 mg/dL lower than in IR patients (190.7 ± 41.8 vs. 173.0 ± 40.0 mg/dL or -9.3%, MD=17.76, 2.89 - 32.64 95%CI, t=2.37, p=0.02) (Table 4). The proportion of patients achieving T-Chol <200 mg/dL was significantly associated with improvement in both groups, but more so with XR: 75% of XR patients were under 200 mg/dL (up from 67% at baseline), versus 63% of IR patients (up from 50% at baseline). The distribution of cholesterol categories (desirable <200 mg/dL, borderline 200-239 mg/dL, high ≥240 mg/dL) shifted favorably in both groups. ii) Triglycerides (TG): baseline median TG was 160.0 (84.8) mg/dL in the IR group and 121.0 (110.0) mg/dL in the XR group. After 6 months, the median TG was significantly reduced in the IR group (145.5 (84.0) mg/dL) and slightly elevated in the XR group (143.0 (96.0) mg/dL), with no statistically significant difference. (IR: median change -14 mg/dL or -8.8%, Z=-1.97, p=0.048; XR: median change +22 mg/dL or +15.4%, Z=-1.94, p=0.052) (Table 2 and Table 3). The distribution of TG levels (normal <150, borderline 150-199, high ≥200 mg/dL) was associated with marginal improvement in both: for example, the percentage with TG ≥150 mg/dL decreased from 61% to 45% in IR (p=0.012) and 46% to 47% in XR (p=0.225). So, metformin IR had a neutral-to-slight TG-lowering effect, and XR did not show a statistically significant advantage over IR for triglycerides. In the between-groups comparison, no statistically significant difference was noted after 6 months of follow-up (145.5 (84.0) vs. 143.0 (96.0) mg/dL, Z=-1, p=0.317) (Table 4).

**Table 2:** within-group change in lipid profile and body measurements with metformin IR

Variable	Metformin IR n=62		95% CI	t/Z	P-value
	Baseline	6 Months			
Lipid profile					
**T-Chol (mg/dL)**	202.4 ± 54.2	190.7 ± 41.8	2.50 – 20.89	2.54	**0.014**
**TG (mg/dL)**	160.0 (84.8)	145.5 (84.0)		-1.97*	**0.048**
**HDL-C (mg/dL)**	41.0 (8.5)	39.5 (8.3)		-0.59*	**0.554**
**LDL-C (mg/dL)**	124.0 ± 47.0	116.9 ± 36.0	-1.09 – 15.24	1.73	**0.088**
Body measurements					
**Body weight (kg)**	82.9 ± 17.7	81.6 ± 18.5	0.17 – 2.41	2.30	**0.025**
**BMI (kg/m^2^)**	**30.9 ± 5.7**	**30.4 ± 5.9**	**0.09 – 0.93**	**2.44**	**0.017**

Significant level at P<0.05; paired sample t-test was adopted in this statistical analysis for parametric data with mean ± SD; * Wilcoxon signed-rank test was adopted in this statistical analysis for non-parametric data with median (IQR)

**Table 3:** within-group change in lipid profile and body measurements with metformin XR

Variable	Metformin XR n=57		95% CI	t/Z	P-value
	Baseline	6 Months			
Lipid profile	-	-	-	-	-
**T-Chol (mg/dL)**	187.7 ± 43.6	173.0 ± 40.0	7.45 – 21.92	4.07	<0.001
**TG (mg/dL)**	121.0 (110.0)	143.0 (96.0)		-1.94*	0.052
**HDL-C (mg/dL)**	41.9 ± 6.3	40.8 ± 7.1	-0.45 – 2.48	1.39	0.172
**LDL-C (mg/dL)**	112.1 ± 43.2	102.3 ± 37.5	3.75 – 15.98	3.23	0.002
Body measurements	-	-	-	-	-
**Body weight (kg)**	84.6 ± 12.8	84.2 ± 13.8	-0.55 – 1.39	0.87	0.389
**BMI (kg/m^2^)**	30.0 ± 4.6	29.8 ± 4.7	-0.19 – 0.56	0.99	0.329

Significant level at P<0.05; paired sample t-test was adopted in this statistical analysis for parametric data with mean ± SD; * Wilcoxon signed-rank test was adopted in this statistical analysis for non-parametric data with median (IQR)

**Table 4:** between-groups change in lipid profile and body measurements over 6 months with metformin IR vs XR

Variable	Metformin IR n=62	Metformin XR n=57	MD	95% CI	t/Z	P-value
Lipid profile	**-**	**-**	**-**	**-**	**-**	**-**
**T-Chol (mg/dL)**	190.7 ± 41.8	173.0 ± 40.0	17.76	2.89 – 32.64	2.37	**0.020**
**TGs (mg/dL)**	145.5 (84.0)	143.0 (96.0)			-1.00*	**0.317**
**HDL-C (mg/dL)**	39.5 (8.2)	40.0 (10.5)			-0.59*	**0.558**
**LDL-C (mg/dL)**	116.9 ± 36.0	102.3 ± 37.5	14.63	1.27 – 27.98	2.17	**0.032**
Body measurements	-	-	-	-	-	-
**Body weight (kg)**	81.6 ± 18.5	84.2 ± 13.8	-2.58	-8.55 – 3.39	-0.86	**0.394**
**BMI (kg/m^2^)**	**30.4 ± 5.9**	**29.8 ± 4.7**	**0.54**	**-1.41 – 2.49**	**0.55**	**0.583**

Significant level at P<0.05; independent sample t-test was adopted in this statistical analysis for parametric data with mean ± SD; * Mann-Whitney U test was adopted in this statistical analysis for non-parametric data with median (IQR)

This is consistent with many findings that metformin's effect on TG is variable and often minimal. iii) HDL cholesterol: the IR group HDL-C median was similar after 6 months (41.0 (8.5) to 39.5 (8.3) mg/dL on average, -1.5 mg/dL or -3.7%, Z=-0.59, p=0.554), and the XR group HDL-C mean was also comparable (41.9 ± 6.3 to 40.8 ± 7.1 mg/dL on average, -1.1 mg/dL or -2.6%, -0.45 - 2.48 95%CI, t=1.39, p=0.172) (Table 2 and Table 3). Neither change was statistically significant, and there was no difference between groups in HDL change (39.5 (8.2) vs. 40.0 (10.5) mg/dL, Z=-0.59, p=0.558) (Table 4). The proportion of patients with very low HDL (<40 mg/dL) was similar in both arms (p=157). iv) LDL cholesterol: the differential effect on LDL-C was even more striking. The IR group's mean LDL-C level decreased from 124.0 ± 47.0 to 116.9 ± 36.0 mg/dL, representing a -7.1 mg/dL or -5.7% change (-1.09 -15.24 95%CI, t=1.73, p=0.088). Meanwhile, the XR group's mean LDL-C dropped from 112.1 ± 43.2 to 102.3 ± 37.5 mg/dL, a substantial reduction of -9.8 mg/dL or -8.7% (3.75 - 15.98 95%CI, t=3.23, p=0.002) (Table 2 and Table 3). By 6 months, the between-group contrast was significant: XR patients experienced an average 12.5% greater decrease in LDL-C than IR patients. Mean LDL-C was ~15 mg/dL lower in the XR group compared to IR (102.3 ± 37.5 vs. 116.9 ± 36.0 mg/dL, MD=14.63, 1.27 - 27.98 95%CI, t=2.17, p=0.032) (Table 4). Clinically, this translated into more XR patients meeting LDL-C targets: 56% of XR patients achieved LDL-C <100 mg/dL, versus only 32% of IR patients (p=0.048). Furthermore, the proportion with LDL-C ≥100 mg/dL decreased drastically from 51% to 44% in the XR group but remained ~68% in the IR group at follow-up. The categorical distribution difference (optimal <100, above optimal 100-129, borderline high 130-159, high 160-189, very high ≥ 190) between groups was statistically significant (p<0.05), underscoring that the XR formulation provided a meaningful LDL-C reduction in this cohort.

In summary, metformin XR showed a clear benefit in reducing total and LDL cholesterol compared to IR in our study. Metformin IR, on its own, did not significantly move LDL or total cholesterol levels in 6 months, whereas XR achieved notable reductions. Triglyceride and HDL outcomes were similar for both formulations, indicating no formulation-specific effect. The results suggest that the XR formulation may provide a lipid profile advantage, particularly in the atherogenic LDL component.

**Changes in body weight and BMI after 6 months:** a key finding of our study is that both metformin IR and XR were weight-neutral over the 6 months, with no significant difference in weight or BMI outcomes between them. At baseline, mean body weight was 82.9 ± 17.74 kg (IR) vs. 84.6 ± 12.8 kg (XR). After 6 months, the mean weight was 81.6 ± 18.5 kg in the IR group and 84.2 ± 13.8 kg in the XR group. These represent minimal changes from baseline: IR group had an average weight change of -1.3 kg or -1.6% (essentially minimal change, 0.17 - 2.41 95%CI, t=2.3, p=0.025), and the XR group had an average change of -0.4 kg or -0.005% (-0.55 - 1.39 95%CI, t=0.87, p=0.389) (Table 2 and Table 3). In the between-groups comparison, no statistically significant difference was noted after 6 months of follow-up (81.6 ± 18.5 vs. 84.2 ± 13.8 kg, MD=-2.58, -8.55 - 3.39 95%CI, t=-0.86, p=0.394) (Table 4).

BMI likewise showed negligible shifts: IR baseline BMI mean 30.9 ± 5.7 kg/m^2^ dropped to 30.4 ± 5.9 kg/m^2^ (-0.5 kg/m^2^ or -2%, 0.09 - 0.93 95%CI, t=2.44, p=0.017); XR baseline BMI mean 30.0 ± 4.6 kg/m^2^ changed to 29.8 ± 4.7 kg/m^2^ (-0.2 kg/m^2^ or -0.007%, -0.19 - 0.56 95%CI, t=0.99, p=0.329) (Table 2 and Table 3). There was no significant difference in weight or BMI change between IR and XR groups (30.4 ± 5.9 vs. 29.8 ± 4.7 kg/m^2^, MD=0.54, -1.41 - 2.49 95%CI, t=0.55, p=583) (Table 4). The consistency of weight outcomes is further reflected in the stability of BMI categories. The proportion of patients classified as obese (BMI ≥30) remained virtually similar from baseline to 6 months in IR (53% to 52%, p=206) and XR (53% to 56%, p=0.655). Several patients in both groups reported lifestyle adjustments (diet improvements, etc.) during their initial diabetes management, which may have prevented weight gain and were even linked to slight weight loss for some, but those effects were evenly distributed.

These findings confirm that metformin did not cause weight gain in our population (a favourable attribute compared to insulin secretagogues or insulin therapy). Moreover, metformin XR did not differ from IR in terms of weight impact, supporting the existing consensus in the literature. The slight weight decrease seen in a fraction of patients could be associated with improvement in glycemic control (less glucosuria and dehydration) or mild appetite suppression from metformin. Some participants on XR had fewer GI side effects than typically seen with IR, but apparently that did not translate into weight differences (if anything, severe GI effects might cause weight loss, but we did not see such differences because few had severe GI issues in either group).

## Discussion

This study suggests insights into the comparative effects of immediate-release (IR) and extended-release (XR) metformin on lipid profile and body measurements in Saudi patients with type 2 diabetes. The most notable finding is that metformin XR was associated with a significantly greater reduction in total and LDL cholesterol over 6 months compared to metformin IR in a cohort of Saudi patients. In contrast, both formulations had equivalent effects on triglycerides, HDL cholesterol, body weight, and BMI, where essentially no differences were observed. These outcomes largely align with and augment existing knowledge of metformin's metabolic effects.

The LDL-C reduction with XR (~15 mg/dL) is approximately 12.5% greater than with IR (~15 mg/dL) and is consistent with Morgan *et al*. who found XR lowered LDL-C by ~5.7 mg/dL compared to IR across trials [14]. This enhanced effect may be due to XR's pharmacokinetics, which prolong hepatic exposure to metformin, thereby enhancing AMPK activation, suppressing cholesterol synthesis, and upregulating LDL receptors [6,15]. Moreover, XR is absorbed more distally in the gut, potentially increasing GLP-1 levels [16], which may indirectly aid lipid metabolism [17,18]. Another plausible factor is adherence. XR's once-daily or twice-daily dosing and better GI tolerability may enhance compliance, ensuring more consistent therapeutic exposure [19,20]. Missing one dose of IR could lead to a 33% reduction in daily dose, possibly explaining why XR patients achieved closer to the intended metformin exposure and, consequently, better lipid outcomes. These pragmatic advantages, rather than purely pharmacologic ones, may underlie XR's superior LDL-C reduction. From a clinical standpoint, a ~15 mg/dL reduction in LDL-C could lower cardiovascular risk, especially in moderate-risk diabetic patients. In this study, 56% of XR patients achieved LDL-C <100 mg/dL, compared with 32% in the IR group, highlighting XR's potential to help more patients reach lipid targets and possibly reduce the need for lipid-lowering medications. Although XR is not a statin substitute, it may serve as a useful adjunct.

Over the past two decades, metformin has been shown to modestly improve the lipid profile, primarily by reducing LDL-C. This study supports the notion that XR retains and possibly enhances this benefit. While genetic or behavioural factors may play a role, our results align with meta-analyses such as Tarry-Adkins *et al*. which found that XR consistently led to greater LDL-C reductions than IR [20]. Derosa *et al*. and Fujioka *et al*. similarly found XR to be more effective than IR in LDL-C lowering [21,22], suggesting that XR's benefits extend across populations. However, some studies offer contrasting results. Schwartz *et al*. observed greater LDL-C reduction with IR [20], while others, such as Gao *et al*. Aggarwal *et al*. Levy *et al*. Fujioka *et al*. Abrilla *et al*. Alshadfan *et al*. and Ghorpade *et al*. reported no significant differences between the formulations [8,10,13,23-26]. This variation likely stems from differences in population characteristics, duration, or study power.

Total cholesterol reduction mirrored LDL-C trends. The XR group showed a greater mean drop in LDL-C (-14.7 mg/dL) than the IR group (-11.7 mg/dL), driven primarily by LDL-C changes, with HDL-C remaining unchanged. XR's superior effect was statistically significant (p<0.001), reinforcing the observed lipid benefits. Our findings on total cholesterol are consistent with Derosa *et al*. who observed superior T-Chol reduction and improved patient satisfaction with XR [21]. Fujioka *et al*. also showed greater lipid improvements with XR [22]. However, others, such as Gao *et al*. Schwartz *et al*. and Aggarwal *et al*. reported no difference [10,20,24]. Levy *et al*. found that XR achieved similar lipid outcomes to IR at lower doses [27], and Fujioka *et al*. reported comparable results after switching formulations [8]. Notably, some meta-analyses, such as Tarry-Adkins *et al*. found no overall difference in total cholesterol between IR and XR [20]. However, Abrilla *et al*. concluded IR may offer greater T-Chol reduction [13]. These inconsistencies highlight population heterogeneity, sample sizes, and study designs as potential factors influencing outcomes.

Regarding triglycerides, XR showed no significant effect, while IR had a slight reduction, consistent with prior studies suggesting metformin's TG-lowering effect is modest (~10%), especially in patients with high baseline TG [7,28]. Our data showed only a slight, non-significant advantage for IR. IR's higher peak concentrations might inhibit lipolysis postprandially more acutely than XR [28], yet this seems clinically negligible. Hence, neither formulation is suitable as primary TG-lowering therapy. Our TG findings align with studies by Gao *et al*. and Tarry-Adkins *et al*. who found no significant differences between formulations [20,24]. However, Derosa *et al*. and Ghorpade *et al*. reported greater TG reduction with XR [21,25], while others, such as Schwartz *et al*. Levy *et al*. and Fujioka *et al*. found TG increases in both groups or more in XR [8,20,22,26]. Aggarwal *et al*. and Abrilla *et al*. concluded IR was more effective for TG reduction [10,13]. These contrasting results likely reflect baseline TG variability and differing dietary practices.

For HDL-C, no significant change was observed in either group, consistent with the majority of the literature. Metformin's limited effect on HDL-C may be due to its modest lipid-modifying potency without statin co-therapy. Studies by Gao *et al*. Schwartz *et al*. and Levy *et al*. similarly found no difference in HDL-C between formulations [20,24,26]. Aggarwal *et al*. observed a slightly larger HDL-C increase with IR (3.4 mg/dL) versus XR (1.6 mg/dL), though the clinical significance is limited [10]. Conversely, Abrilla *et al*. reported significantly improved HDL-C with IR [13], though such effects may not generalise broadly.

Turning to body weight and BMI, our findings reinforce metformin's weight neutrality, regardless of formulation. Over 6 months, IR users experienced a significant weight loss of an average of only 1.3 kg, while no weight difference was noted in XR users. A few individuals in each group lost a small amount of weight, often those who were obese or overweight, and experienced an improved diet or mild gastrointestinal side effects initially. However, the differences between groups were overall not significant (p=0.394). These data confirm previous reports, such as Abrilla *et al*. and Gao *et al*. that both formulations yield comparable weight and BMI outcomes [13,24]. Metformin's expected modest weight effect (2-3 kg over 1-2 years) likely wasn't fully captured during the 6-month window [29,30]. Also, as patients were newly diagnosed, initial metabolic adjustments (rehydration, reduced osmotic diuresis) may have neutralised early weight changes [31]. Some suggest that XR may cause fewer GI side effects and therefore less early-on appetite suppression, but our data did not support a difference in weight loss between groups. Patients typically adapt to GI effects within weeks, reducing their long-term impact on weight. Clinically, these findings suggest that while metformin can modestly aid weight control, other interventions (e.g., diet, GLP-1 receptor agonists) are needed for significant weight loss. The choice of IR vs. XR won't significantly affect weight trajectory, so it should be based on tolerability and glycemic/lipid considerations rather than weight.

Literature comparing IR and XR weight outcomes is mixed. Levri *et al*. found no evidence that metformin reduces weight [32], whereas Ning *et al*. found significant weight and BMI reductions [33]. Our findings align with multiple studies showing no formulation-based weight differences [10,13,24,25,34]. A meta-analysis by Tarry-Adkins *et al*. found no meaningful difference in weight change, and post-treatment BMI was identical across formulations [20]. Alshadfan *et al*. also reported no significant differences in BMI or waist circumference [28]. One RCT in non-diabetic obese adolescents using XR vs. placebo found modest reductions in BMI, reinforcing that XR is effective but not superior to IR for weight [35]. The convergence of evidence supports the assertion that both IR and XR are equivalent with respect to body weight outcomes, and that any differences, if present, are not clinically significant. From a practice perspective, XR may be preferred in patients with borderline LDL-C requiring better lipid control, especially if tolerability is a concern. Though not a substitute for statins, XR can complement lipid management. Patients who find IR hard to tolerate due to GI symptoms can switch to XR without losing glucose or weight benefits.

It is important to acknowledge that this was an observational study. Differences in lipid outcomes could be influenced by unmeasured confounders, such as diet or physical activity. Although both groups received the same counselling, better adherence and overall wellness in XR users (due to fewer side effects and simpler dosing) may have led to better lipid responses. The systematic random sampling and similar baseline characteristics suggest comparability across groups. Additionally, the lack of weight differences supports the idea that any dietary differences did not significantly bias results. Another factor is the slight sample size imbalances (62 vs. 57), and unmeasured factors (like baseline diet patterns) may theoretically affect outcomes, but the strength and consistency of LDL-C reduction argue for a real, pragmatic benefit of XR beyond random variation.

## Conclusion

This study highlights that the choice of metformin formulation in managing type 2 diabetes can have implications beyond glycemic control. Metformin extended-release (XR) was associated with a greater improvement in key lipid parameters. Specifically, total cholesterol and LDL cholesterol, compared to the immediate-release (IR) formulation, over 6 months. Patients on metformin XR achieved significantly lower LDL cholesterol levels than those on IR, suggesting a potential added cardiovascular benefit to using the XR formulation. In contrast, responses to triglycerides and HDL cholesterol were similar between XR and IR, and both formulations preserved metformin's hallmark weight-neutral effect, with equivalent effects on body mass index. The absence of a statistically or clinically significant difference in body weight between formulations reinforces their equivalence in terms of body weight impact. For clinicians, these findings indicate that metformin XR can be selected not only for its convenience and tolerability but also with the expectation of achieving equal or better improvements in the patient's lipid profile. Particularly in patients where LDL cholesterol is a concern, metformin XR may be the preferred formulation, as it can modestly enhance LDL cholesterol reduction alongside standard lipid-lowering measures. At the same time, one can be confident that switching from IR to XR will not adversely affect weight control (a critical consideration in obese diabetic patients) and will maintain the same glycemic efficacy. In conclusion, metformin XR is associated with weight outcomes comparable to IR, with the added benefit of slightly more favourable changes in total and LDL cholesterol levels. This advantage, although modest, reinforces the role of metformin XR as a valuable component of comprehensive diabetes care, targeting not just blood glucose but also components of metabolic syndrome. Future research could investigate the long-term cardiovascular outcomes of patients on metformin XR versus IR; however, our results already support the preferential use of XR in appropriate patients to maximise the metabolic benefits of metformin therapy.

**Limitations:** several limitations should be noted. First, the study was observational and not randomised, introducing potential selection bias. Although baseline characteristics (including lipids and BMI) were well-matched between groups, unmeasured factors (such as dietary habits or changes in physical activity) could have influenced lipid outcomes. Second, the 6-month follow-up is relatively short for evaluating changes in weight and lipids, which might take longer to fully manifest. However, significant differences in LDL-C were observed even in this time frame. Third, our sample size, particularly for subgroup analyses, was moderate; a larger sample would provide greater power to detect small differences in HDL-C or TG. Fourth, we did not control for or record the use of lipid-modifying supplements or dietary changes in detail (patients were advised uniformly), but individual adherence to diet recommendations could vary. We excluded patients who started statins during the study; however, it's possible that some patients made lifestyle changes that improved their lipid levels. Fifth, although our findings align with international data, all patients were from a single centre in Saudi Arabia, which may limit the generalizability of our results to the kingdom as a whole and to other ethnic or geographic populations. Finally, we measured lipids at only two time points; more frequent monitoring might have provided a clearer picture of the trajectory and demonstrated variability.

### What is known about this topic

- Metformin modestly improves lipid parameters (especially triglycerides and LDL cholesterol) primarily through AMPK activation and improved insulin sensitivity;
- Extended-release (XR) formulations are better tolerated gastrointestinally and may enhance adherence compared to immediate-release (IR);
- IR and XR forms have similar glycemic effects, but their impact on lipid profiles and body composition remains less clearly defined in real-world settings.

### What this study adds

- Demonstrates that metformin XR leads to significantly greater reductions in total and LDL cholesterol compared to IR, suggesting enhanced lipid benefits independent of glycemic control;
- Confirms that both IR and XR formulations have equivalent effects on BMI, supporting XR's weight neutrality despite being associated with improvement in tolerability;
- Supports the clinical use of XR metformin not just for adherence and GI tolerability, but also for its modest yet meaningful advantage in lipid profile improvement, particularly LDL-C reduction.
